# Ganglioside GM2, highly expressed in the MIA PaCa-2 pancreatic ductal adenocarcinoma cell line, is correlated with growth, invasion, and advanced stage

**DOI:** 10.1038/s41598-019-55867-4

**Published:** 2019-12-18

**Authors:** Norihiko Sasaki, Kenichi Hirabayashi, Masaki Michishita, Kimimasa Takahashi, Fumio Hasegawa, Fujiya Gomi, Yoko Itakura, Naoya Nakamura, Masashi Toyoda, Toshiyuki Ishiwata

**Affiliations:** 1 Research Team for Geriatric Medicine (Vascular Medicine), Tokyo Metropolitan Institute of Gerontology, Tokyo, 173-0015 Japan; 2 Department of Pathology, Tokai University School of Medicine, Kanagawa, 259-1193 Japan; 3 Department of Veterinary Pathology, School of Veterinary Medicine, Nippon Veterinary and Life Science University, Tokyo, 180-8602 Japan; 4 Division of Aging and Carcinogenesis, Research Team for Geriatric Pathology, Tokyo Metropolitan Institute of Gerontology, Tokyo, 173-0015 Japan

**Keywords:** Pancreatic cancer, Pancreatic cancer

## Abstract

Gangliosides, a group of glycosphingolipids, are known to be cell surface markers and functional factors in several cancers. However, the association between gangliosides and pancreatic ductal adenocarcinoma (PDAC) has not been well elucidated. In this study, we examined the expression and roles of ganglioside GM2 in PDAC. GM2+ cells showed a higher growth rate than GM2− cells in the adherent condition. When GM2– and GM2+ cells were cultured three-dimensionally, almost all cells in the spheres expressed GM2, including cancer stem cell (CSC)-like cells. A glycolipid synthesis inhibitor reduced GM2 expression and TGF-β1 signaling in these CSC-like cells, presumably by inhibiting the interaction between GM2 and TGFβ RII and suppressing invasion. Furthermore, suppression of GM2 expression by MAPK inhibition also reduced TGF-β1 signaling and suppressed invasion. GM2+ cells formed larger subcutaneous tumors at a high incidence in nude mice than did GM2– cells. In PDAC cases, GM2 expression was significantly associated with younger age, larger tumor size, advanced stage and higher histological grade. These findings suggest that GM2 could be used as a novel diagnostic and therapeutic target for PDAC.

## Introduction

With an overall survival rate of ~8%, pancreatic cancer is one of the most lethal human malignancies. Surgical treatment offers the only possible cure for pancreatic ductal adenocarcinoma (PDAC), a major histological type of pancreatic cancer, but 80% of all PDAC patients are inoperable at diagnosis due to the advanced stage of the cancer^1^. Chemotherapies or chemoradiotherapies can reduce tumor size and improve prognosis, but these treatments do not completely eliminate PDAC cells in patients. Since most PDAC patients are over 60 years old, the rapid increase of aging populations worldwide is likely to have a corresponding increase in incidence of PDAC. By 2030, pancreatic cancer is expected to become the second-leading cause of cancer-related deaths in USA^2^. Hence, development of methods for detecting PDAC at earlier stages and effective therapies for advanced PDAC patients are urgently required.

Recent studies have shown that cancerous tumors consist of heterogeneous populations of cancer cells, including a small number of cancer stem cells (CSCs)^3,4^. CSCs are believed to be correlated with tumor initiation, growth, and metastasis, and are resistant to chemotherapies and chemoradiotherapies. It has been suggested that elimination of CSCs could lead to the disappearance of cancer cells, based on the concept that CSCs are responsible for tumor self-renewal^5^. Therefore, a better understanding of the molecular mechanisms of PDAC carcinogenesis and metastasis is needed to identify potential tumor biomarkers and to develop effective therapeutic strategies for metastasis and recurrence of PDAC.

Glycosphingolipids, which are composed of sugar chains attached to the sphingolipid ceramide, have been used as cell surface markers and are also known to regulate several biological functions^6–8^. Gangliosides, which are molecules composed of glycosphingolipids linked with one or more sialic acids, are known to be key components of lipid rafts, which act as platforms for signal transduction^9,10^. Changes in ganglioside levels affect the expression of raft-associated proteins on the cell surface and lead to reduced membrane fluidity, resulting in cellular dysfunctions, such as impaired signal transduction^11–14^. The ganglioside GM2, which is one of the major series of gangliosides, has several biological functions, such as cell adhesion and signal transduction^15,16^. GM2 is highly expressed in several types of human malignant tumors, such as melanomas, gliomas, and neuroblastomas, but are absent or weakly expressed in normal tissues^17,18^. Furthermore, highly metastatic murine tumor cells contain higher amounts of gangliosides, including GM2, than non-metastatic tumor cells^19^. GM2 also plays crucial roles in metastasis in human malignant melanoma and small-cell lung cancers^20,21^.

Thus, GM2 is functionally important and is expected to be a therapeutic target in some types of cancers. However, neither the roles nor the expression levels of GM2 in pancreatic cancer have been elucidated. In this study, we showed expression of GM2 in human PDAC cells and PDAC tissues, and identified that GM2 is correlated with growth, invasion and advanced stage of PDAC. Our results suggest that GM2 could be a novel diagnostic and therapeutic target for some types of pancreatic cancer.

## Results

### GM2 is expressed on the cell surface in PDAC cell lines

**Figure 1:**
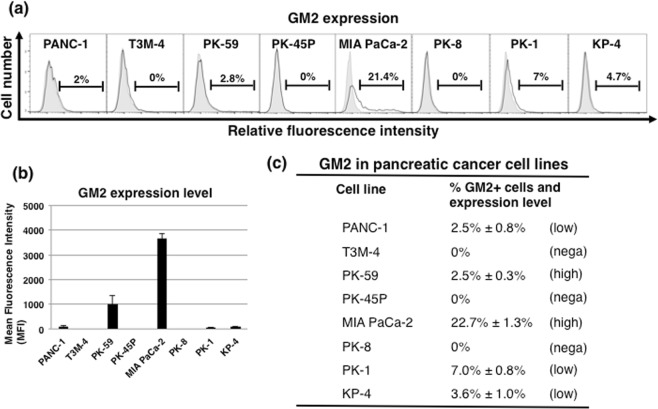
The expression of GM2 in human PDAC cell lines. (**a**) FACS analysis of GM2 expression in several PDAC cell lines cultured in adherent conditions. Controls are indicated by thin lines with gray color. (**b**) Levels of GM2 expression in several PDAC cell lines. Mean fluorescence intensities (MFIs) relative to those of PANC-1 cells are shown. (**c**) Classification of PDAC cell lines into negative, low and high GM2 expression based on FACS analysis. Intensity of GM2 expression is denoted as high/low based on the MFI. High indicates >1000 MFI; low indicates 20–100 MFI; “nega” indicates negative staining.

First, fluorescence-activated cell sorter (FACS) analysis using GM2-specific antibody was performed to examine the expression of GM2 in eight human PDAC cell lines (PANC-1, T3M-4, PK-59, PK-45P, MIA PaCa-2, PK-8, PK-1 and KP-4). In adherent-cultured human PDAC cells, the fraction of GM2+ cells was higher (21.4%) in MIA PaCa-2 than in the other cell lines (Fig. 1a). GM2+ cells were less than 10% in other PDAC cell lines, and were not detected in T3M-4, PK-45P or PK-8. Furthermore, the intensity of GM2 expression was also highest in MIA PaCa-2, with PK-59 showing the second highest GM2 expression intensity (Fig. 1b). Based on these findings, the eight PDAC cell lines were classified into 3 groups: negative for GM2 expression (T3M-4, PK-45P, PK-8), GM2 low expression (PANC-1, PK-1, KP-4) and GM2 high expression (MIA PaCa-2, PK-59) (Fig. 1c). Due to its high GM2 + expression, further *in vitro* and *in vivo* experiments were performed using MIA PaCa-2 cells.

### There were no notable morphological differences between GM2– and GM2+ cells in adherent culture conditions

**Figure 2:**
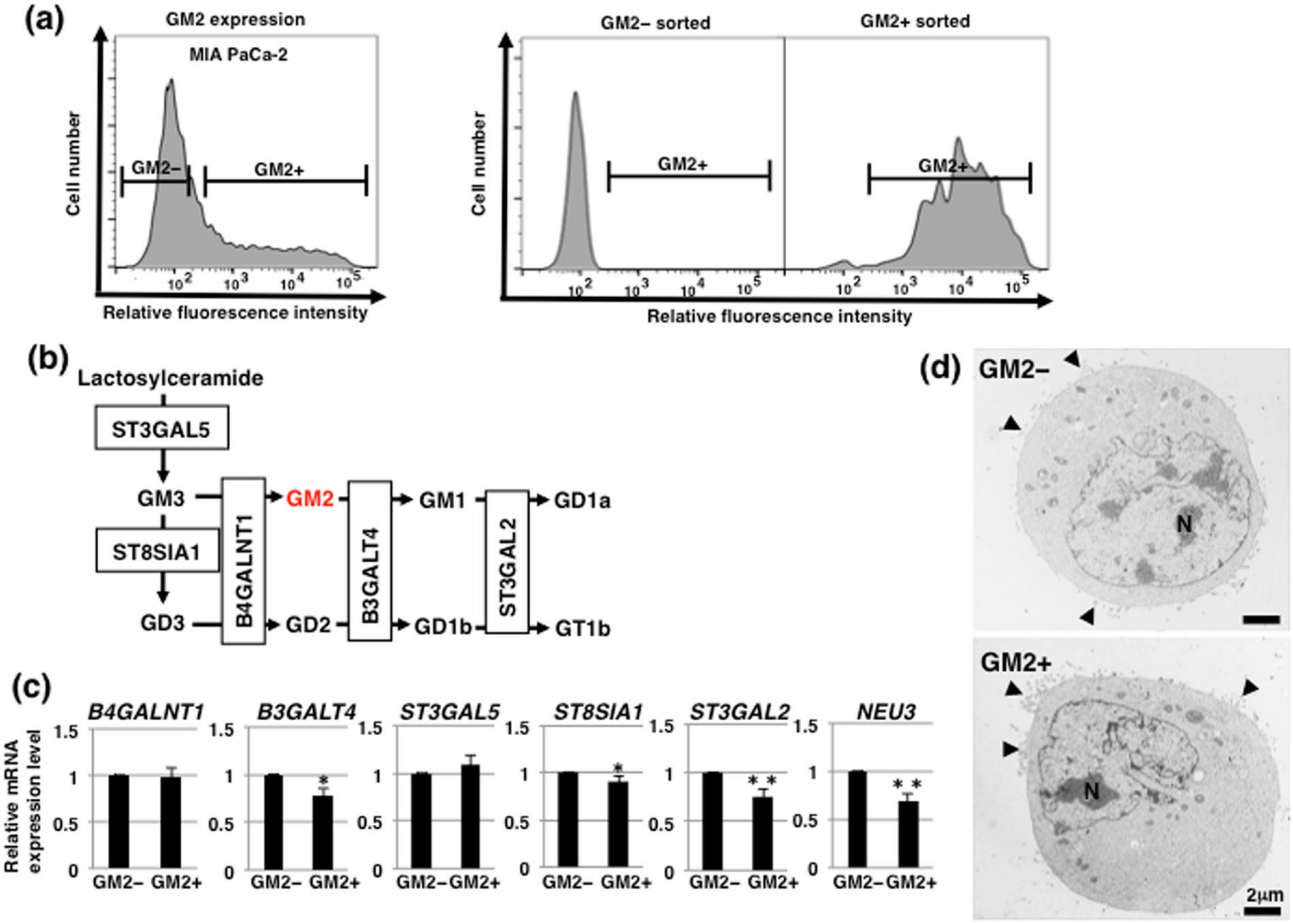
Morphological analysis of GM2– and GM2+ cells in adherent culture. (**a**) Sorting of GM2– and GM2+ cellsGM2+ cells in MIA PaCa-2. GM2 expression in MIA PaCa-2 before sorting is shown in the left panel. Levels of GM2 in MIA PaCa-2 after sorting were re-analyzed by flow cytometry (right panel). The gate represents GM2+ cells. (**b**) Main synthetic pathway of gangliosides. GM2 is shown in red. Glycosyltransferases contributing to each synthetic pathway are also shown. (**c**) Real-time PCR analysis of the glycosyltransferases shown in b and NEU3 in GM2– and GM2+ cells. Results shown are normalized to values obtained for GM2– cells (value = 1). *p < 0.05, **p < 0.01. (**d**) TEM analysis in GM2– and GM2+ cells. Microvilli and nuclei are indicated by arrowheads and the letter N, respectively.

To compare the characteristics of GM2– and GM2+ cells, we sorted MIA PaCa-2 based on GM2 expression level. FACS-reanalysis of sorted cells showed that the fraction of GM2+ cells in cells sorted from GM2 negative or positive populations were approximately 0% (GM2– populations) or 95% (GM2+ populations), respectively (Fig. 2a). These reanalyzed results confirm that the GM2– and GM2+ cells were well isolated. As shown in Fig. 2b, GM2 expression is regulated by the action of glycosyltransferases and/or sialidase (NEU3), which is a plasma membrane-associated sialidase that modulates ganglioside content by removing sialic acid. To elucidate the molecules that contribute to GM2 expression in GM2+ cells, we analyzed the expression levels of the glycosyltransferases and *NEU3*. Real-time PCR analysis showed that levels of *ST8SIA1*, *B3GALT4*, *ST3GAL2* and *NEU3* expression were lower in GM2+ cells than in GM2– cells (Fig. 2c). Next, we compared morphology between GM2– and GM2+ cells. There were no remarkable morphological differences between GM2– and GM2+ cells apparent from phase contrast microscopy (data not shown). Transmission electron microscopy (TEM) was used to investigate morphology in detail, showing that both GM2– and GM2+ cells developed microvilli (arrowheads) on cell surface and had large nucleoli (N) (Fig. 2d). No notable morphological differences were observed between GM2– and GM2+ cells at the ultramicroscopic level.

### GM2+ cells in adherent culture conditions exhibit high growth rates and are highly sensitive to anti-cancer drugs

**Figure 3:**
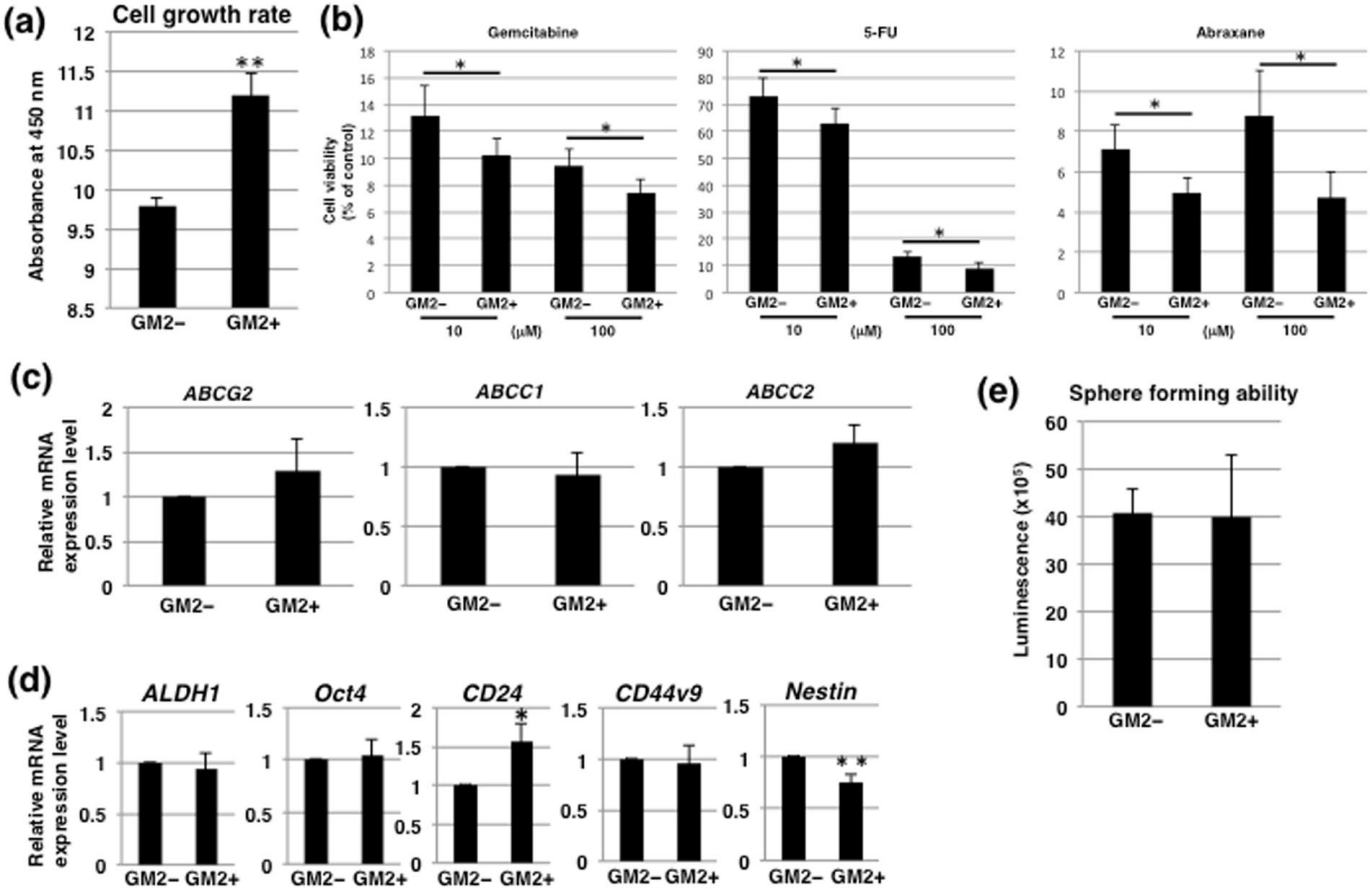
Comparison of cell growth, stemness, and anti-cancer drug resistance in GM2– and GM2+ cells cultured in adherent conditions. (**a**) Comparison of cell growth rates in adherent culture. The cell growth rate of GM2+ cells was significantly higher than that of GM2– cells. (**b**) Anti-cancer drug resistance assay in GM2– and GM2+ cells. The dose response (10 or 100 μM) of GM2– and GM2+ cells to gemcitabine, 5-FU, and abraxane was determined using the ATP assay. (**c**) Real-time PCR analysis of transporters in GM2– and GM2+ cells. Results are normalized to values obtained for GM2– cells (value = 1). *ABCB1* did not have a detectable signal. (**d**) Real-time PCR analysis of stemness markers in GM2– and GM2+ cells. Results shown are normalized to values obtained for GM2– cells (value = 1). *p < 0.05, **p < 0.01. (**e**) Sphere forming assays performed in GM2– and GM2+ cells.

We also investigated the functional properties of GM2+ cells. The rate of GM2+ cell growth in adherent conditions was significantly higher than that of GM2– cells (Fig. 3a). The effects of three commonly used anti-pancreatic cancer drugs, gemcitabine, 5-FU, and abraxane, on GM2+ cells were evaluated to determine the CSC properties of the cells. CSCs possess an effective efflux pathway that confers resistance to anticancer drugs. The survival rates of GM2+ cells were lower than those of GM2– cells after treatment with the anticancer drugs at either 10 or 100 μM (Fig. 3b). Furthermore, we examined the levels of expression of four genes encoding potential anticancer drug transporters. Real-time PCR analysis revealed that expression of *ABCG2*, *ABCC1*, and *ABCC2* were not significantly different between GM2– and GM2+ cells (Fig. 3c). We further examined stemness of GM2+ cells using real-time PCR analysis of CSC markers. Of the markers assayed, only *CD24* had higher levels of expression in GM2+ cells than in GM2– cells, while *Nestin* was lower in GM2+ cells (Fig. 3d). Another method commonly used to examine CSC characteristics, especially self-renewal ability under the floating condition^4^, is the sphere formation assay. ATP assays showed that the number of cells in the spheres was not different in GM2 + and GM2– cells (Fig. 3e), indicating no differences in sphere-forming capability between the two types of cells. Hence, GM2+ cells in adherent culture conditions exhibited high growth rates and were highly sensitive to anti-cancer drugs but did not have remarkable stem cell characteristics compared with GM2– cells.

### GM2 is expressed in CSC-like sphere cells induced by 3D culture conditions

**Figure 4:**
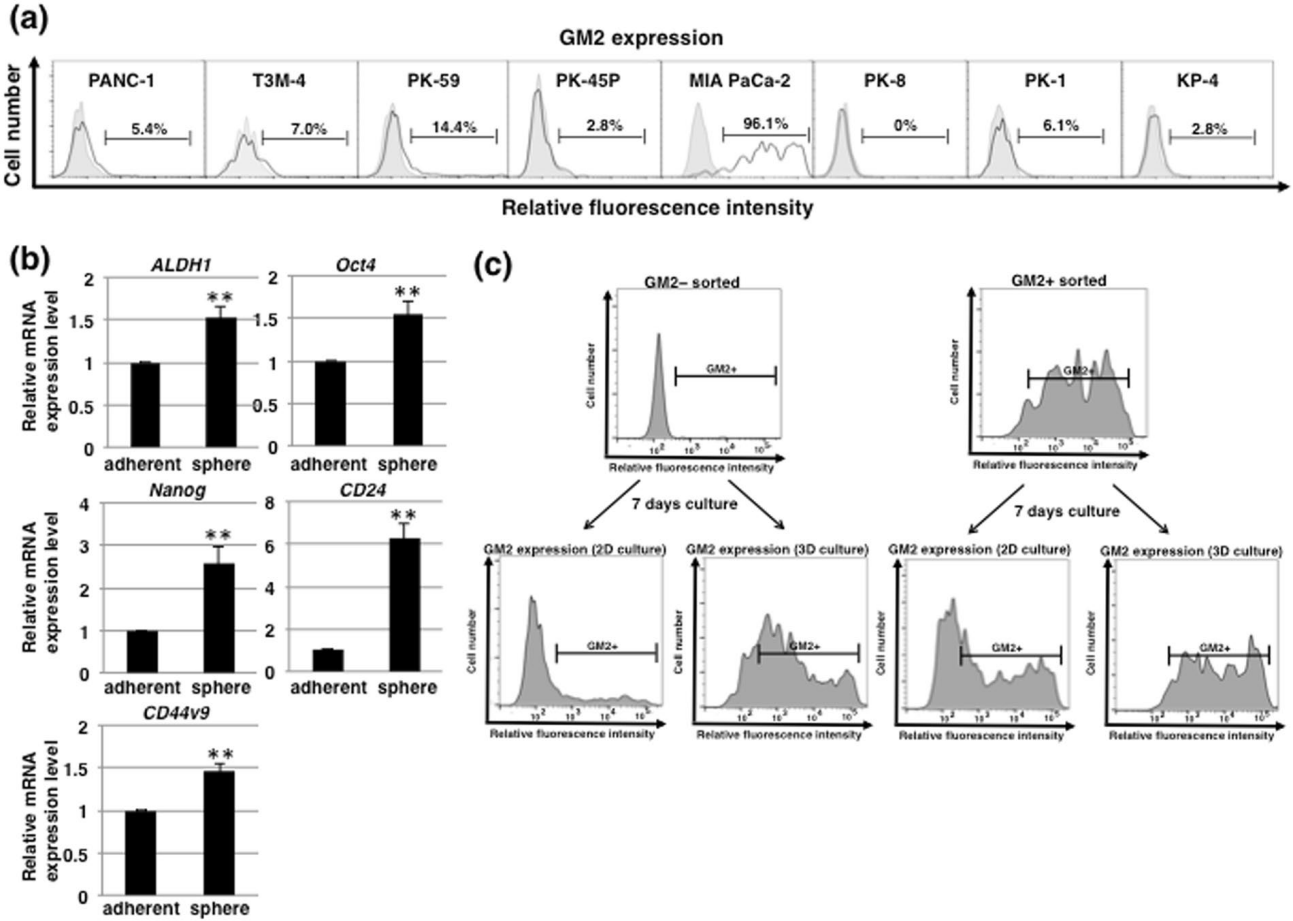
Expression of GM2 in CSC-like cells induced by 3D culture conditions. (**a**) FACS analysis of GM2 expression in several PDAC cell lines cultured in 3D conditions. Controls are indicated by thin lines with gray color. (**b**) Real-time PCR analysis of stemness markers in MIA PaCa-2 cultured in adherent or 3D conditions. Results shown are normalized to values obtained for adherent-cultured cells (value = 1). **p < 0.01. (**c**) FACS analysis of GM2 expression in 7 days-cultured cells after sorting of adherent cultured MIA PaCa-2.

We recently reported that pancreatic CSCs can be enriched *in vitro* using anchorage-independent 3D culture^22,23^ and employed this technique to further investigate the relationship between GM2 expression and stem cells. FACS analysis of GM2 after 3D culture showed that almost all cells in the spheres expressed GM2 in MIA PaCa-2 (96.1%) whereas the fraction of GM2+ cells in other cell lines were not significantly changed, except in PK-59 in which GM2+ cells were still less than 15% of cells in the spheres (Fig. 4a). Real-time PCR analysis of stemness markers in MIA PaCa-2 showed that each stemness markers were increased in sphered cells compared with adherent-cultured cells (Fig. 4b). These results suggest that CSC-like MIA PaCa-2 cells induced in 3D culture expressed GM2. We next examined the potential for GM2 synthesis in sorted GM2– and GM2+ cells in adherent culture conditions (Fig. 2a) after re-culture under adherent or 3D culture. Under adherent culture, the expression pattern of GM2 in both sorted GM2– and GM2+ cells returned to the steady state of unsorted adherent-cultured cells after 7 days of re-culture (Fig. 4c). By contrast, sorted GM2+ and GM2– cells expressed high levels of GM2 after 7 days of re-culture under 3D culture (Fig. 4c). Thus, 3D culture induced GM2+ CSC-like cells and GM2– cells have potential to synthesize GM2 and did so in 3D culture conditions.

### GM2+ CSC-like cells exhibit responsiveness against TGF-β1 via interaction with GM2 and TGF-receptor

**Figure 5:**
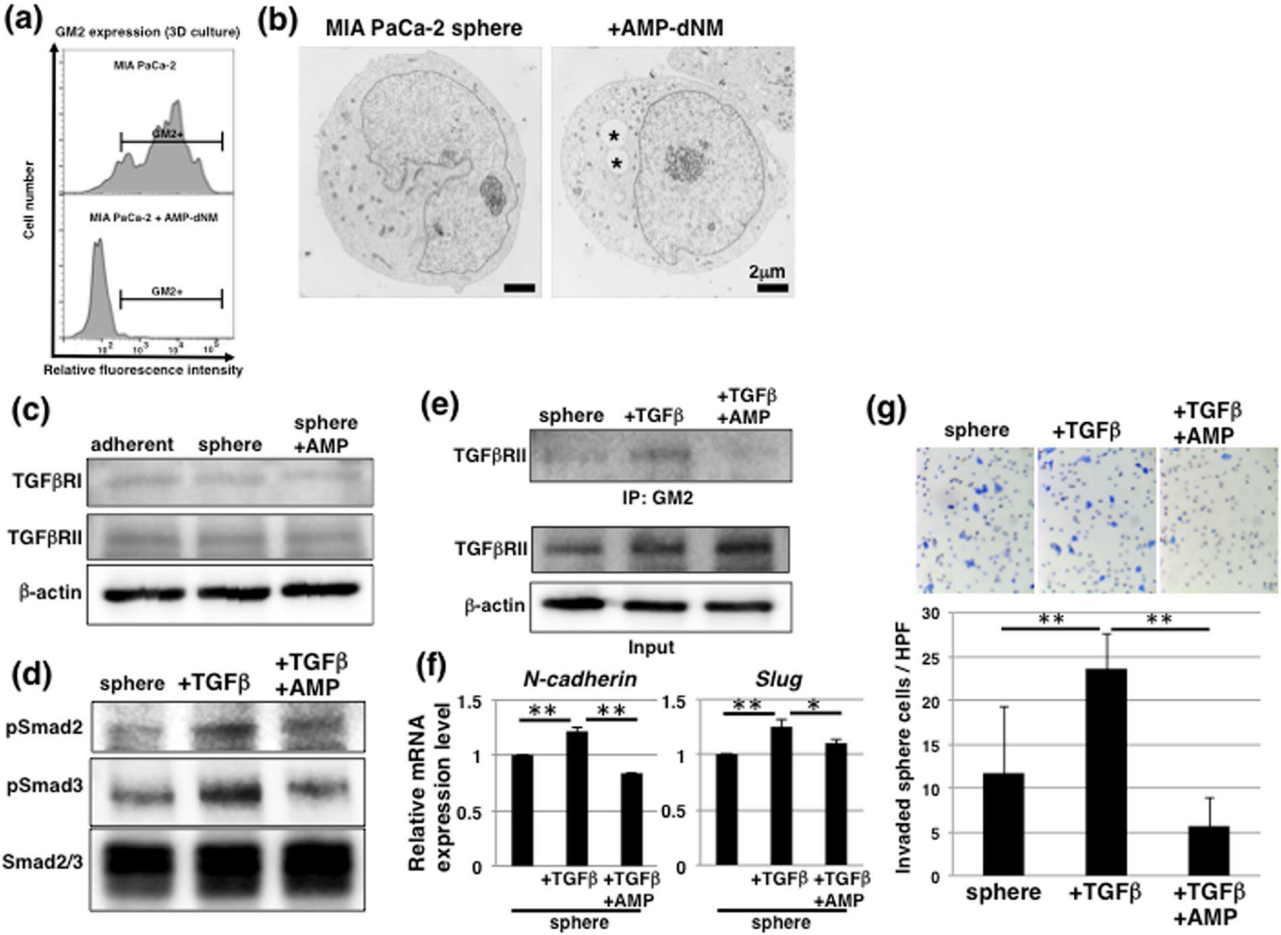
TGF-β1 signaling via interaction between GM2 and TGFβRII in GM2+ CSC-like cells. (**a**) FACS analysis of GM2 expression in MIA PaCa-2 cultured in 3D conditions with or without 10 μM AMP-dNM treatment. (**b**) TEM analysis of MIA PaCa-2 cultured in 3D conditions with or without 10 μM AMP-dNM treatment. Asterisks indicate vacuoles. (**c**) Immunoblotting for TGFβRI and TGFβRII in adherent, sphere, and AMP-dNM-treated sphere cells. The blot images were cropped to highlight the TGFβRI, TGFβRII and β-actin bands using the anti-TGFβRI, anti-TGFβRII and anti-β-actin antibodies. The full blot images are presented in the Supplementary western blot dataset. (**d**) Immunoblotting for TGF-β1 signaling in sphere cells with or without AMP-dNM treatment. Seven days after sphere formation, sphere cells were cultured for an additional 2 days with or without 10 ng/ml TGF-β1. The blot images were cropped to highlight the pSmad2, pSmad3 and Smad2/3 bands using the anti-pSmad2, anti-pSmad3 and anti-Smad2/3 antibodies. The full blot images are presented in the Supplementary western blot dataset. (**e**) Immunoblotting performed in the immunoprecipitates with anti-GM2 antibody for TGFβRII (upper panel). Input images are shown in the lower panel. The blot images were cropped to highlight the TGFβRI, TGFβRII and β-actin bands using the anti-TGFβRI, anti-TGFβRII and anti-β-actin antibodies. The full blot images are presented in the Supplementary western blot dataset. (**f**) Real-time PCR analysis of EMT markers in sphere cells. Results shown are normalized to values obtained for non-treated sphere cells (value = 1). *p < 0.05, **p < 0.01. (**g**) Matrigel invasion assays performed in sphere cells. **p < 0.01.

Next, to investigate the role of GM2 in GM2+ CSC-like MIA PaCa-2 cells, we used *N*-(5’-adamantane-1’-yl-methoxy)-pentyl-1-deoxynojirimycin (AMP-dNM), which is a specific inhibitor of glucosylceramide synthase that can be used to study the functional roles of endogenous gangliosides without affecting ceramide levels^24,25^. Treatment with AMP-dNM lowered GM2 expression and that of other gangliosides (GM3, GM1, and GD1a) on sphere cells (Fig. 5a and S1). Morphological analysis of control and AMP-dNM-treated MIA PaCa-2 sphere cells using TEM showed that there were few microvilli on the surface of sphere cells and that vacuoles (asterisks) were noticeable in AMP-dNM-treated cells (Fig. 5b). The growth of sphere cells was not affected by AMP-dNM treatment (Fig. S2A), and real-time PCR analysis showed that expression of stemness markers in sphere cells was not affected by AMP-dNM treatment (Fig. S2B). Furthermore, anti-cancer drug resistance determined by ATP assays showed that anti-cancer drug resistance was higher in sphere cells and was accompanied by increased expression of transporters (*ABCG2* and *ABCC2*) compared with adherent-cultured cells and were not negatively affected in sphere cells treated with AMP-dNM (Fig. S3). These results suggest that CSC-like cells enriched in 3D culture conditions have high GM2 expression, but that GM2 does not functionally affect stemness.

Epithelial-mesenchymal transition (EMT) has recently been reported to be associated with CSCs and malignant behaviors in cancer^26–28^. We investigated whether GM2 + sphere cells were responsive against TGF-β1, an EMT inductive factor, and could further the contribution of GM2 to TGF-β1 signaling. Western blot analysis showed that TGFβRI and TGFβRII in sphere cells were expressed to the same extent as in adherent-cultured cells, regardless of AMP-dNM treatment (Fig. 5c), and that activation of TGF-β1 signaling, as indicated by phosphorylation of Smad2/3 in TGF-β1-treated sphere cells, was attenuated by AMP-dNM treatment (Fig. 5d). These results suggest that GM2 contributes to the regulation of TGF-β1 signaling. TGF-β1 signaling occurs dynamically after interaction between TGFβRI and TGFβRII, which are localized in the lipid rafts^29^. GM2 is also located on lipid rafts complexed with integrin and modulates the downstream signaling pathway^30^. Immunoprecipitation analysis was performed to confirm the involvement of GM2 in TGF-β1 signaling. Interaction of GM2 and TGFβRII was observed in TGF-β1-treated sphere cells and this interaction was inhibited by AMP-dNM treatment (Fig. 5e). Furthermore, expression of EMT markers, such as *N-cadherin* and *Slug*, in conjunction with activation of TGF-β1 signaling was observed (Fig. 5f). Furthermore, increased invasion in TGF-β1 treated sphere cells was also suppressed by AMP-dNM treatment, determined using invasion assays with matrigel-coated chamber (Fig. 5g). Taken together, these data suggest that expression of GM2 was linked to CSCs, and that GM2 correlated with regulation of TGF-β1 induced EMT and is involved in invasiveness of MIA PaCa-2 cells.

### GM2 expression in 3D culture conditions is regulated by MAPK pathway

**Figure 6:**
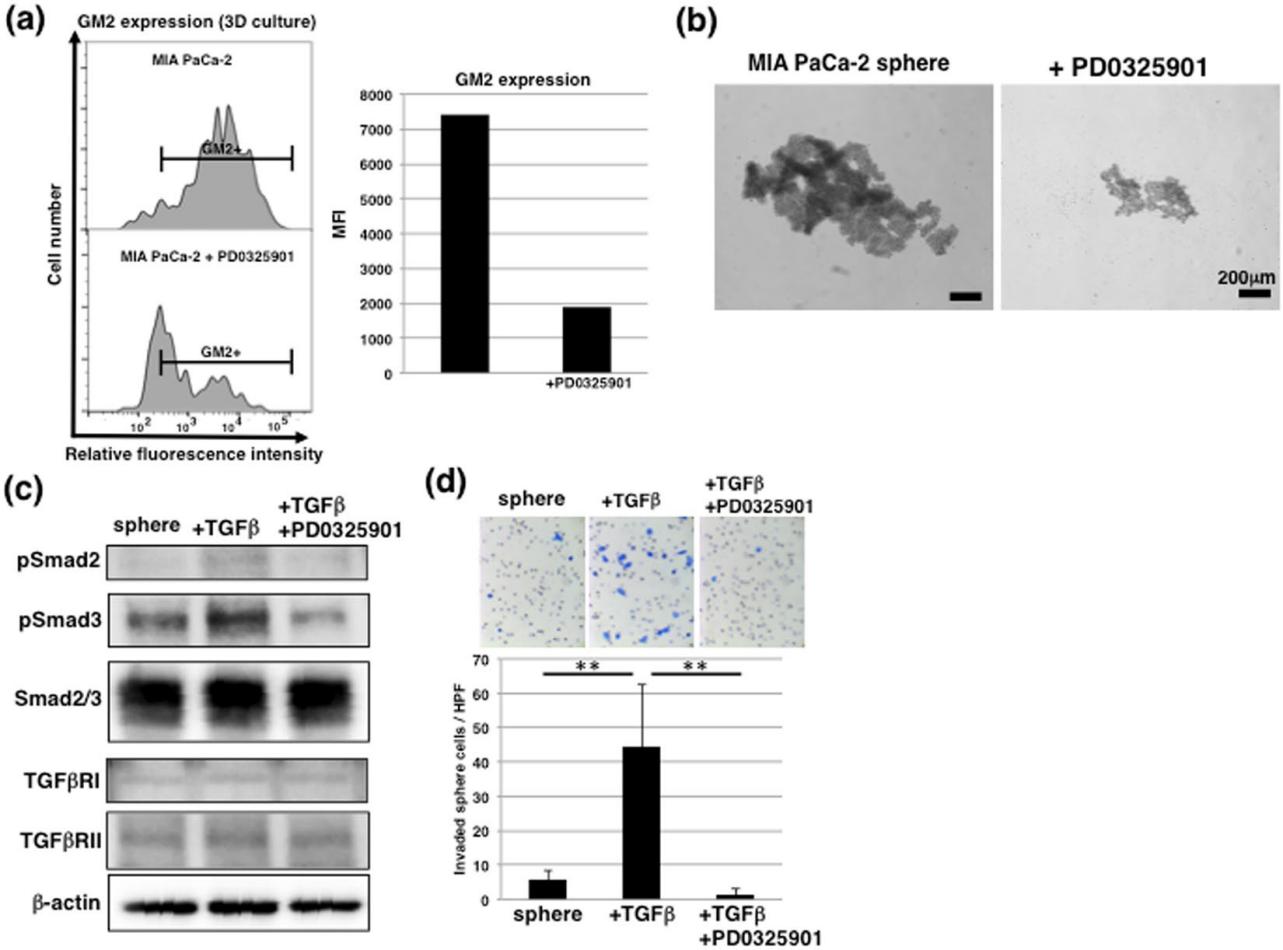
Regulation of GM2 expression in CSC-like cells by MAPK pathway. (**a**) FACS analysis of GM2 expression in MIA PaCa-2 cultured in 3D conditions with or without 1 μM PD0325901 treatment. Mean fluorescence intensities (MFIs) are shown on the right side. (**b**) Phase contrast images of MIA PaCa-2 cultured in 3D conditions with or without 1 μM PD0325901 treatment. (**c**) Immunoblotting for TGF-β1 signaling in sphere cells with or without 1 μM PD0325901 treatment. Seven days after sphere formation, sphere cells were cultured for an additional 2 days with or without 10 ng/ml TGF-β1. The blot images were cropped to highlight the pSmad2, pSmad3, Smad2/3, TGFβRI, TGFβRII and β-actin bands using the anti-pSmad2, anti-pSmad3, anti-Smad2/3, anti-TGFβRI, anti-TGFβRII and anti-β-actin antibodies. The full blot images are presented in the Supplementary western blot dataset. (**d**) Matrigel invasion assays performed in sphere cells. **p < 0.01.

To clarify the contribution of GM2 expression to signaling in CSC-like cells, we examined GM2 expression after treatment with inhibitors of several signaling pathways that are considered to be involved in sphere formation and CSC self-renewal, such as the FGF/EGF and JAK/STAT3 pathways^31,32^. Among signal inhibitors, we found that PD0325901 (MEK inhibitor) decreased GM2 expression in sphere cells, a decrease that was accompanied by a reduction of sphere size (Fig. 6a,b). Next, we used real-time PCR to examine whether glycosyltransferases and/or NEU3 were associated with GM2 synthesis, finding that *NEU3* expression was downregulated and all others were increased in sphere cells compared with adherent-cultured cells (Fig. S4). By contrast, PD0325901 treatment significantly decreased *ST3GAL5* expression in sphere cells compared with non-treated sphere cells (Fig. S4). As shown in Fig. 2b, ST3GAL5 contributes to synthesis of all gangliosides at the end of ganglioseries. This suggests that inhibition of GM2 in sphere cells via PD0325901 may be dependent on attenuated expression of *ST3GAL5*. Furthermore, PD0325901 treatment attenuated activation of TGF-β1 signaling (Fig. 6c) and further inhibited increases in invasion of TGF-β1-treated sphere cells (Fig. 6d). These results suggest that suppression of GM2 expression by treatment with an MEK inhibitor suppressed the increased invasion associated with activation of TGF-β1 signaling in CSC-like cells.

### GM2+ cells exhibit higher tumor forming ability than GM2– cells in nude mice

**Figure 7:**
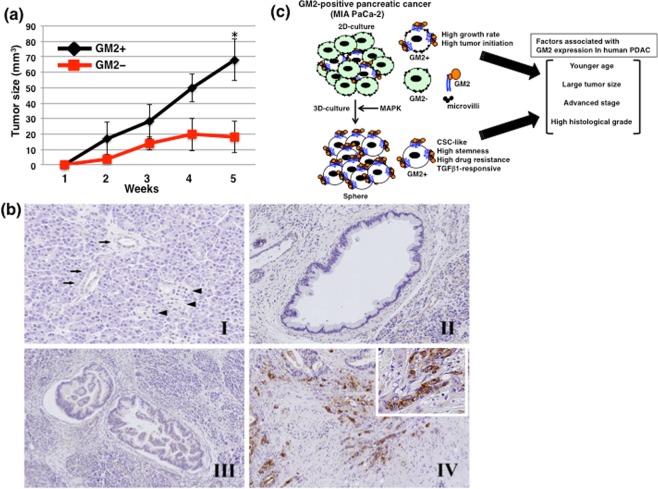
Heterotopic implantation of GM2– or GM2+ cells into nude mice and localization in human PDAC cells. (**a**) Transplantation of GM2– or GM2+ cells into nude mice. GM2–/+ sorted MIA PaCa-2 cells were transplanted subcutaneously into BALB/cAJcl-nu/nu mice. Tumor formation and size were observed 5 weeks after transplantation. *p < 0.05. (**b**) Representative photographs of immunohistochemistry for GM2. Normal pancreatic duct (arrows) was negative or faintly positive for GM2. Islet cells (arrowheads) and acinar cells are negative for GM2 (I). The foci of low-grade (II) and high-grade (III) pancreatic intraepithelial neoplasia are negative for GM2. Strong membranous and/or cytoplasmic expression of GM2 is observed in human PDAC cells (IV, inset). (**c**) A hypothetical model of GM2 expression in pancreatic cancer.

To determine whether GM2+ cells have the same tumor-initiating potential as GM2– cells *in vivo*, sorted GM2– and GM2+ cells (Fig. 2a) were subcutaneously transplanted into BLAB/c nude mice. GM2+ cells generated more tumors than GM2– cells in nude mice (5 tumors/5 mice vs 3 tumors/5 mice). The average size of tumors derived from GM2+ cells was larger than from GM2– cells and the difference was statistically significant at 5 weeks (68.0 ± 13.5 mm3 vs 18.1 ± 10.3 mm3, *p* < 0.05) (Fig. 7a).

### GM2 is expressed in human PDAC cases

In order to clarify the expression and roles of GM2 in human PDAC cases, we performed immunohistochemical analyses on 117 human pancreas tissues. Normal pancreatic ducts in the PDAC tissues were negative or faintly positive for GM2. Islet cells and acinar cells were also negative for GM2. GM2 was positive in 18.8% (n = 22) of PDAC cases (Fig. 7b and Table 1). In pancreatic intraepithelial neoplasia (PanIN) lesions close to PDAC cells, GM2 was positive in 2.6% (n = 1) of low-grade PanIN cases and positive in 3.6% (n = 1) of high-grade PanIN cases. The number of cases with GM2 expression was significantly higher in PDAC tissues than in low-grade and high-grade PanIN (*p* = 0.01). There were no significant differences in GM2 expression between low-grade PanIN and high-grade PanIN (low grade PanIN vs high-grade PanIN, *p* = 0.672).

**Table 1:** GM2 expression and clinicopathological parameters.

	GM2 expression		*p* - value
	Positive	Negative	
	*n* (%)	*n* (%)	
**PanIN and PDAC**			
Low-grade PanIN (*n* = 38)	1 (2.6)	37 (97.4)	0.01
High-grade PanIN (*n* = 28)	1 (3.6)	27 (96.4)	
PDAC (*n* = 117)	22 (18.8)	95 (81.2)	
**PDAC cases**			
**Age**			
= < 65 years (*n* = 44)	13 (29.5)	31 (70.5)	0.021
>65 years (*n* = 73)	9 (12.3)	64 (87.7)	
**Gender**			
Male (*n* = 60)	12 (20)	48 (80)	0.734
Female (*n* = 57)	10 (17.5)	47 (82.5)	
**Primary tumor (pT)**			
pT1-pT2 (*n* = 83)	16 (19.3)	67 (80.7)	0.838
pT3 (*n* = 34)	6 (17.6)	28 (82.4)	
**Regional lymph node metastasis (pN)**			
pN0 (*n* = 50)	6 (12)	44 (88)	0.104
pN1-2 (*n* = 67)	16 (23.9)	51 (76.1)	
**Distant metastasis (pM)**			
pM0 (*n* = 113)	22 (19.5)	91 (80.5)	0.429
pM1 (*n* = 4)	0	4 (100)	
**Stage**			
Stage IA-IB (*n* = 38)	3 (7.9)	35 (92.1)	0.036
Stage IIA–IV (*n* = 79)	19 (24.1)	60 (75.9)	
**Size**			
<50 mm (*n* = 95)	21 (22.1)	74 (77.9)	0.045
=> 50 mm (*n* = 22)	1 (4.5)	21 (95.5)	
**Perineural invasion**			
No (*n* = 9)	1 (11.1)	8 (88.9)	0.466
Yes (*n* = 108)	21 (19.4)	87 (80.6)	
**Lymphatic involvement**			
No (*n* = 10)	3 (30)	7 (70)	0.282
Yes (*n* = 107)	19 (17.8)	88 (82.2)	
**Venous involvement**			
No (*n* = 8)	1 (12.5)	7 (87.5)	0.535
Yes (*n* = 109)	21 (19.3)	88 (80.7)	
**Tumor grade**			
G1 or G2 (*n* = 64)	7 (10.9)	57 (89.1)	0.017
G3 (*n* = 53)	15 (28.3)	38 (71.7)	

PanIN, pancreatic intraepithelial neoplasia; PDAC, pancreatic ductal adenocarcinoma.

Next, we examined correlations between GM2 expression and clinicopathological factors. GM2 expression was significantly associated with younger patients (65 years; *p* = 0.021), larger tumor size (≧50 mm; *p* = 0.045), advanced TNM stage (stage IIA–IV; *p* = 0.036), and advanced histological grade (G3; *p* = 0.017). Conversely, no significant associations were observed between GM2 expression and gender, perineural invasion, lymphatic involvement, venous involvement, pT factor, pN factor, or pM factor (Table 1). There was no correlation between GM2 expression and overall patient survival (*p* = 0.628).

## Discussion

Several gangliosides have previously been identified in CSCs^32^. For example, ganglioside GD2 was identified as a CSC marker in breast cancer^33^. Reduction of GD2 by inhibition of GD3 synthase in breast cancer cells reduced the CSC population and CSC-associated properties, including *in vivo* tumor formation^33^. Another report showed that GD2, GD3, GM2, and GD1a are highly expressed in breast CSCs, but only GD2 and GD3 were suggested to contribute to sphere formation and cell motility^34^. In the current study, we report for the first time that GM2 are highly expressed in pancreatic CSC-like cells. Although the association between GM2 with pancreatic CSCs is unknown, GM2 is associated with metastasis in melanoma and lung carcinoma^20,21^. Here we found that GM2 may be involved in invasion of the adjacent extracellular matrix through regulation of TGF-β1 signaling in pancreatic CSCs. High invasiveness to stromal tissues of GM2+ cells may lead to larger tumor size in animal experiments and human PDAC tissues. Future investigations focusing on GM2, *e.g*. using anti-GM2 antibodies, may lead to new PDAC therapies targeting GM2. Downregulation of NEU3, a plasma membrane-associated sialidase that modulates ganglioside content by removing sialic acid, is known to contribute to increases in GM2 expression^35^. As shown in this study, *NEU3* expression is reduced in adherent cultured GM2+ cells compared with GM2– cells and in CSC-like cells, indicating that the level of NEU3 expression may be involved in regulation of GM2 expression. It is necessary to further clarify what factors are involved in the expression of GM2 in CSC-like cells.

In this study, we demonstrated that AMP-dNM treatment could inhibit TGF-β1 signaling and invasion, presumably by inhibiting the interaction between GM2 and TGFβRII. It has been previously reported that AMP-dNM treatment improves glucose tolerance, reduces hepatic steatosis, and enhances insulin response in rodent models of type 2 diabetes^25,36^. Furthermore, AMP-dNM treatment was demonstrated to reduce the development of atherosclerosis by lowering plasma cholesterol levels in APOE*3-Leiden and low-density lipoprotein receptor -/- mice^37^. Thus, the effects of AMP-dNM on age-related diseases, including diabetes and arteriosclerosis, are known at the individual level. Incidence of pancreatic cancer is also increased in the aged population, and changes in glycosylation that occur with aging are thought to correlate with carcinogenesis and tumor progression. Therefore, the suppressive effects of AMP-dNM on tumor invasion to stromal tissues may be expected in pancreatic cancer, which is frequently seen in PDAC.

Several monoclonal antibodies (mAbs), such as the chimeric anti-EGFR mAb cetuximab, have been successfully introduced into clinical practice to treat cancer patients^38^. For example, it has been demonstrated that humanized anti-GM2 antibodies inhibited production of multiple organ metastases and prolonged survival of the SCID mouse in a metastasis model induced by GM2-expressing small-cell lung cancer cells^21^. GM2 is predominantly detected in poorly differentiated types of pancreatic cancer and correlates with growth and invasion, therefore therapeutics using anti-GM2 antibodies may be expected to have potential in treatment of pancreatic cancer. On the other hand, it has been shown that anchorage-independent growth and lung metastasis in triple-negative breast cancer were inhibited by MEK inhibitors^39^. In the present study, we showed for the first time that treatment with an MEK inhibitor attenuated GM2 synthesis in pancreatic CSC-like cells, suppressed anchorage-independent growth and increased invasion associated with activation of TGF-β1 signaling. Further study will be required, but the MEK inhibitor, which acts on MAPK signaling, may have clinical potential for preventing invasion in GM2 + pancreatic cancer cases.

It is known that gangliosides are secreted from cancer cells by shedding and have pleiotropic effects, such as regulation of tumor growth^40^, angiogenesis^41,42^, and immune modulation^43^. The exosome contains a cell membrane antigen, including ganglioside^44^, so the exosome-expressed ganglioside would also be secreted into the blood. Furthermore, it has been reported that GM2 can be quantified in plasma using LC/ESI-MS/MS^45^, a method that could be used to detect GM2 derived from cancers in the blood. In the current study, prominent GM2 expression was observed in MIA PaCa-2 cells (established from poorly differentiated cells obtained from a PDAC patient), in poorly differentiated human pancreatic cancer tissues and in pancreatic CSC-like cells. Hence, there is the possibility that shed GM2 and/or exosome bound GM2 could be detected in the blood of patients with early stage, poorly differentiated pancreatic cancer.

In conclusion, we showed that a PDAC cell line expressing GM2 in 2D-culture exhibited a high growth rate and high tumor initiation, pancreatic CSC-like cells expressing GM2 in 3D-culture exhibit responsiveness against TGF-β1 resulting in promotion of invasion, and GM2 expression is associated with growth and advance of human PDAC (Fig. 7c). Therefore, further study will be required for development of an early detection method for GM2 expressing pancreatic cancers and novel therapeutic strategies targeting GM2.

## Methods

### Cell culture

Cell culture was performed as previously described^46,47^. The human PDAC cell lines PK-1, PANC-1, PK-59, and MIA PaCa-2 were obtained from the Cell Resource Center for Biomedical Research, Institute of Development, Aging and Cancer, Tohoku University (Sendai, Japan). PK-45P, PK-8, T3M-4, and KP-4 human PDAC cells were provided by the RIKEN BRC through the National Bio-Resource Project of the MEXT, Japan. Cells were grown in growth medium (RPMI 1640 medium containing 10% fetal bovine serum) at 37 °C under a humidified 5% CO_2_ atmosphere. For EMT induction, cells were cultured for 48 h in growth medium containing 10 ng/mL TGF-β1 (Peprotech, Rocky Hill, NJ, USA). For 3D culture, cells in growth medium were plated at 1.0 × 10^4^ cells/well or 3.0 × 10^3^ cells/well in 24-well ultra-low attachment plates (Corning Inc. Kennebunk, ME, USA) or 96-well ultra-low attachment plates (Thermo Fisher Scientific, Waltham, MA, USA), respectively. The spheres were aspirated after 7 days using micropipettes and placed in microcentrifuge tubes for use in further experiments. Photographs of the spheres were taken using a sphere analyzing device, Cell3iMager duos (SCREEN Holdings Co., Ltd., Kyoto, Japan).

### Cases and tissue samples

A total of 117 cases of PDAC were examined. All patients underwent surgical resection at the Tokai University Hospital (Kanagawa, Japan) between 2007 and 2017. Only cases of conventional PDAC were included in the analysis. Patients who received preoperative chemotherapy or who were diagnosed as special histological types of cancer, such as adenosquamous carcinoma, mucinous carcinoma, undifferentiated carcinoma or invasive intraductal papillary mucinous neoplasm, were excluded. All surgical materials were fixed in formalin and embedded in paraffin. Formalin-fixed and paraffin-embedded (FFPE) tissue samples were cut into 4 μm-thick sections and stained with H&E. Pathological TNM (pT/pN/pM) staging and histological grade were classified according to the Union for International Cancer Control, eighth edition^48^. Overall survival was defined as the time interval between surgery and death or the date of the last patient visit. PanIN contained in the PDAC sections was evaluated. PanIN was classified as either low or high grade^49^. A total of 66 PanIN lesions (38 low grade and 28 high grade) were identified. The mean age of the 117 patients with PDAC was 67.4 years (range 43–86 years), and the cohort included 60 men (51.3%) and 57 women (48.7%). The mean tumor size was 35.8 mm (range 7–110 mm) in diameter. Thirty-two patients (27.4%) had grade 1 (G1; well-differentiated adenocarcinoma) tumors, thirty-two patients (27.4%) had G2 (moderately differentiated adenocarcinoma), and 53 patients (45.3%) had G3 (poorly differentiated adenocarcinoma) tumors. UICC stage pT1b, pT1c, pT2, and pT3 cancers were diagnosed in 2 (1.7%), 16 (8.7%), 65 (55.6%), and 34 (29.1%) patients, respectively. UICC stage pN0, pN1, and pN2 was diagnosed in 50 (42.7%), 48 (41%), and 19 patients (16.2%), respectively. Four patients (3.4%) had distant metastasis. Stage IA, stage IB, stage IIA, stage IIB, stage III, and stage IV disease were diagnosed in 13 (11.1%), 25 (21.4%), 12 (10.3%), 47 (40.2%), 16 (13.7%), and 4 (3.4%) patients, respectively. Intrapancreatic nerve invasion, lymphatic involvement, and venous involvement were found in 108 (92.3%), 107 (91.5%), and 109 (93.2%) patients, respectively.

### FACS analysis and cell sorting

FACS analysis of gangliosides and cell sorting in PDAC cells were performed as previously reported^50,51^. Cells were harvested and dissociated single cells were incubated on ice for 30 min with anti-GM2 antibody (TCI, Tokyo, Japan), anti-ganglioside GM3 (NBT Laboratories, Tokyo, Japan), anti-ganglioside GD1a (TCI), anti-ganglioside GD3 (Merck Millipore), anti-ganglioside GD2 (TCI), and anti-ganglioside GD1b (TCI) diluted in FACS buffer (0.5% [w/v] BSA and 0.1% [w/v] sodium azide in PBS). After washing, the cell suspension was incubated on ice for 30 min with Alexa Fluor® 488-conjugated secondary antibodies (Molecular Probes, Eugene, OR, USA) diluted in FACS buffer. For ganglioside GM1 detection, cells were incubated with Alexa Fluor® 647-conjugated cholera toxin B subunit (Molecular Probes) diluted in FACS buffer for 30 min on ice. Cells were then washed and suspended in cell sort buffer (25 mM HEPES pH 7.0, 1 mM EDTA, 1% BSA in PBS). Cell sorting and analysis were performed using a FACSAria™ Cell Sorter (Becton Dickinson, Franklin Lakes, NJ, USA). Mean fluorescence intensities (MFIs) were calculated by subtracting the intensities of the controls.

### TEM

Cells were prepared for TEM and TEM was performed as previously described^22^. Briefly, cells were fixed with 2.5% glutaraldehyde in 0.1 M phosphate buffer (pH 7.4), then postfixed for 1 h with 2% OsO_4_ dissolved in distilled water, dehydrated in a graded series of ethanol solutions, and embedded in Epon. Ultrathin sections were made on an ultramicrotome and stained with uranyl acetate and lead citrate for TEM (H-7500, Hitachi).

### Real-time PCR

Real-time PCR was performed as previously described^47^. Total RNA was isolated from cells using the RNeasy plus mini kit (QIAGEN, Hilden, Germany) and subsequently reverse-transcribed using the ReverTra Ace® qPCR RT Kit (Toyobo, Osaka, Japan) according to the manufacturer’s instructions. Real-time PCR was performed using the Power Sybr® Green kit (Applied Biosystems, Foster City, CA, USA) and a StepOnePlus™ real-time PCR system (Applied Biosystems). β-actin was amplified as an internal control. Primer sets for real-time PCR are listed in Table S1.

### Cell proliferation assays

Cell proliferation assays were performed as previously described^50^. Cells were cultured in growth medium at a density of 5 × 10^3^ cells/well in 96-well plates followed by incubation for 72 h. Adherent cells were then incubated with WST-8 cell counting reagent (Wako Pure Chemical Industries Osaka, Japan) for 2 h. Optical density was measured at 450 nm using a plate reader (Bio-Rad Laboratories, Hercules, CA). ATP assays were used to examine proliferation in spheres using the CellTiter-Glo® 2.0 Assay (Promega, Madison, USA) according to the manufacturer’s protocol.

### Anti-drug resistance assays

Anti-drug resistance assays were performed as previously described^50^. Cells (3.0 × 10^3^ cells/well) were plated in 96-well culture plates with growth medium. Each anti-cancer drug was administered at the indicated concentration after 1 day and cell growth rates were measured by ATP assays after 3 days. ATP assays were performed 4 days after treatment of anti-cancer drug for determination of drug resistance in spheres. Cell viability was calculated as the percentage of luminescence in drug-treated cells relative to non-treated control cells.

### Sphere forming assays

Sphere forming assays were performed as previously described^47^. Cells (3.0 × 10^3^ cells/well) were cultured in 96-well ultra-low attachment plates with RPMI 1640 medium containing FGF-2 (10 ng/mL, ReproCELL, Tokyo, Japan) and EGF (20 ng/mL, AUSTRAL Biologicals, San Ramon, CA) for 7 days. Sphere proliferation, evaluated by ATP assays, was evaluated to assess sphere forming capability.

### Invasion assays

Invasion assays were performed using Corning matrigel invasion chambers (pore size: 8 μm, Discovery Labware Inc., Bedford, USA) as previously reported^47^. Cells were plated at a density of 1 × 10^5^ cells/500 μl on the upper surface of the inserts, and, 24 h later, cells that had migrated through the membrane to the lower surface of the filter were fixed and stained with a Diff-Quick staining kit (Polysciences, Inc., Warrington, PA, USA) and enumerated under a light microscope.

### Immunoblotting

Immunoblotting and immunoprecipitations were performed as previously described^51^. Cells were lysed with lysis buffer (50 mM Tris-HCl pH 7.4, 150 mM NaCl, 1.5 mM MgCl_2_, 5 mM EDTA, and 1% Triton™ X-100) containing protease and phosphatase inhibitor cocktails and immunoprecipitations were performed with anti-GM2 antibody and protein L magnetic beads (Thermo Fisher Scientific, Waltham, MA, USA). Samples prepared as described above were separated by SDS-PAGE and then transferred onto PVDF membranes (Merck Millipore, Billerica, MA, USA). After blocking, the membranes were incubated with the following primary antibodies: monoclonal rabbit anti-pSmad2 (#3108; Cell Signaling Technology, Danvers, MA, USA), monoclonal rabbit anti-pSmad3 (ab52903; Abcam, Cambridge, UK), monoclonal rabbit anti-Smad2/3 (#8685; Cell Signaling Technology), polyclonal anti-TGFβRI (SAB4502958; Sigma-Aldrich, St. Louis, MO, USA), polyclonal anti-TGFβRII (sc-220; Santa Cruz Biotechnology, Dallas, TX, USA), and monoclonal mouse anti-β-actin (A5316; Sigma-Aldrich). Membranes were then incubated with the appropriate peroxidase-conjugated secondary antibodies (Cell Signaling Technology), washed and developed with ECL™ Prime reagents (GE Healthcare, Piscataway, NJ, USA).

### Heterotopic implantation of PDAC cells

In order to clarify *in vivo* tumorigenicity of GM2– or GM2 + PDAC cells, 1 × 10^5^ cells/animal (n = 5, 5 sites per cell line) were subcutaneously injected into the bilateral flank of nine-week old, female, athymic mice (BALB/cAJcl-nu/nu; CLEA Japan Inc, Tokyo, Japan). Tumor volume was calculated weekly using the formula: volume = a × b^2^ × 0.5, where a is the longest diameter and b is the shortest. The animals were monitored for 5 weeks. All experiments were approved by the Animal Experiments Committee of Nippon Veterinary and Life Science University and were performed in accordance with Guidelines for Animal Experiments by the Nippon Veterinary and Life Science University.

### Immunohistochemistry

Immunohistochemical analysis of GM2 expression was performed by cutting FFPE blocks into 4 μm-thick sections and staining with anti-GM2 antibody (dilution 1:1000; TCI) using BOND-MAX (Leica Biosystems) according to the manufacturer’s instructions. Normal kidney tissue was used as a positive control expression. GM2 expression in PDAC or PanIN cells that was equal to or stronger than that in the positive control was considered positive. The percentage of membranous and/or cytoplasmic cells that stained positively for GM2 in the total PDAC cells or total cells in a single PanIN lesion was evaluated. GM2 expression was considered positive when 5% of the cancer cells in the tissues were stained.

### Ethics statements

All experiments and methods were performed in accordance with relevant guidelines and regulations. Specifically, primary human pancreatic cancer specimens were obtained with consent from patients who were admitted to Tokai University Hospital. All participants provided written informed consent for this investigation. The investigation was performed under approval of the Institutional Review Board at Tokai University (permission number: 17R220) and Tokyo Metropolitan Hospital and Institute of Gerontology (permission number: R17-50). All animal experiments were performed under the approval of the Animal Experiments Committee of Nippon Veterinary and Life Science University (permission number: 30K-27).

### Statistical analysis

Statistical analyses for *in vitro* studies were performed using EZR (Saitama Medical Centre, Jichi Medical University; http://www.jichi.ac.jp/saitama-sct/SaitamaHP.files/statmedEN.html; Kanda, 2012) or Microsoft Excel 2010 (Microsoft Corporation, Redmond, WA, USA). Results are expressed as means ± standard deviation (SD) from three independent experiments. Unpaired Student’s t-test and one-way ANOVA with Tukey’s HSD test were used for comparing two or more groups, respectively. Results of tumor size were expressed as means ± standard error (SE) and a Mann–Whitney test was used. Statistical analyses of immunohistochemistry were conducted using IBM SPSS Statistics software, version 25.0 (IBM, Chicago, IL, USA). Fisher’s exact test or Pearson’s χ2 test was used to analyze the relationship between clinicopathological factors and GM2 expression and the changes in GM2 expression during carcinogenesis in low grade PanIN, high-grade PanIN and PDAC. Overall survival curves and median survival times were plotted using the Kaplan-Meier method, and compared using the log-rank test. All data were representative of at least two independent experiments. *p* values < 0.05 were considered significant.

## Supplementary information

Supplementary material
