# Supplementary material for: Ganglioside GM2, highly expressed in the MIA PaCa-2 pancreatic ductal adenocarcinoma cell line, is correlated with growth, invasion, and advanced stage
Source: Sci Rep. 2019 Dec 18;9:19369. doi: 10.1038/s41598-019-55867-4 (PMC6920443; doi:10.1038/s41598-019-55867-4)
Supplement: Supplementary file 1 — Supplementary material [file 41598_2019_55867_MOESM1_ESM.docx]

**Supplementary Material**

**Ganglioside GM2, highly expressed in the MIA PaCa-2 pancreatic ductal adenocarcinoma cell line, is correlated with growth, invasion, and advanced stage**

Norihiko Sasaki^*^, Kenichi Hirabayashi, Masaki Michishita, Kimimasa Takahashi, Fumio Hasegawa, Fujiya Gomi, Yoko Itakura, Naoya Nakamura, Masashi Toyoda and Toshiyuki Ishiwata^*^

Division of Aging and Carcinogenesis, Research Team for Geriatric Pathology, Tokyo Metropolitan Institute of Gerontology, Tokyo 173-0015, Japan

*Corresponding author:

E-mail:sasanori@tmig.or.jp

E-mail: [tishiwat@tmig.or.jp](mailto:tishiwat@tmig.or.jp)

**Table of Contents:**

1. Supplemental table

2. Supplemental figures

3. Supplementary western blot dataset

**1. Supplementary table**

Table S1. Primer sets used for real-time PCR

| **Gene** | **Forward primer** | **Reverse primer** |
| --- | --- | --- |
| *Oct4* | GGAGGAAGCTGACAACAATGAAA | GGCCTGCACGAGGGTTT |
| *Nestin* | TCCTGCTGTAGATGCAGAGATCAG | ACCCTGTGTCTGGAGCAGAGA |
| *CD24* | TCCAACTAATGCCACCACCAA | GACCACGAAGAGACTGGCTGTT |
| *CD44v9* | AGCAGAGTAATTCTCAGAGCTT | TGCTTGATGTCAGAGTAGAAGT |
| *E-cadherin* | CCAGTGAACAACGATGGCATT | TGCTGCTTGGCCTCAAAAT |
| *N-cadherin* | TGGGAATCCGACGAATGG | GCAGATCGGACCGGATACTG |
| *Slug* | TGCGGCAAGGCGTTTT | TCTCCCCCGTGTGAGTTCTAA |
| *β-actin* | GGTCATCACCATTGGCAATGAG | TACAGGTCTTTGCGGATGTCC |
| *B4GALNT1* | ACAGCAGACACAGTCCGGTTCT | GCGGGTGTCTTATGCGGATA |
| *B3GALT4* | GAAGGAGGCCAGGTTTTGC | CCCGGCCCAAGTACAGAAG |
| *ST3GAL5* | AGGAATGTCGTCCCAAGTTTG | GGAGTAAGTCCACGCTATACCT |
| *ST8SIA1* | TACTCTCTCTTCCCACAGG | GACAAAGGAGGGAGATTGC |
| *ST3GAL2* | TGGACGGGCACAACTTCA | TGCCAACATCCTGCTCAAAG |
| *NEU3* | AATGTGAAGTGGCAGAGGTGA | TCACAGAGCTGTCGACTCAGG |
| *ABCG2* | TGGCTGTCATGGCTTCAGTACT | CATTATGCTGCAAAGCCGTAAA |
| *ABCB1* | TGACAGCTACAGCACGGAAG | TCTTCACCTCCAGGCTCAGT |
| *ABCC1* | GAGAGTTCCAAGGTGGATGC | AGGGCCCAAAGGTCTTGTAT |
| *ABCC2* | TACCAATCCAAGCCTCTACC | AGAATAGGGACAGGAACCAG |

**2. Supplementary figures**

**Figure S1.** FACS analysis of the major gangliosides shown in Fig. 2B in MIA PaCa-2 cells cultured in 3D conditions with or without 10 μM AMP-dNM treatment. Positively expressed gangliosides (GM3, GM1, and GD1a) in sphere cells were downregulated after AMP-dNM treatment. GD3, GD2, and GD1b were not detected in sphere cells.

**Figure S2.** Comparison of sphere cell growth and stemness marker expression between non-treated and AMP-dNM-treated sphere cells. (A) Cell proliferation assays performed in spheres from non-treated and AMP-dNM-treated sphere cells. (B) Real-time PCR analysis of stemness markers in non-treated and AMP-dNM-treated sphere cells.

**Figure S3.** Comparison of anti-cancer drug resistance between non-treated and AMP-dNM-treated sphere cells. (A) Anti-cancer drug resistance assay in adherent-cultured MIA PaCa-2. Dose (10 or 100 μM) response of adherent-cultured MIA PaCa-2 to gemcitabine, 5-FU, and abraxane was determined using the ATP assay. (B) Anti-cancer drug resistance assay in sphere cells with or without AMP-dNM treatment. Dose (10 or 100 μM) response of sphere cells to gemcitabine, 5-FU, and abraxane was determined using the ATP assay. (C) Real-time PCR analysis of transporters in adherent- or 3D-cultured cells with or without AMP-dNM treatment. Results shown are normalized to values obtained for adherent-cultured cells (value = 1). **p* < 0.05, ***p* < 0.01. n.s.: not significant.

**Figure S4.** Real-time PCR analysis of glycosyltransferase encoding genes *B4GALNT1, B3GALT4, ST3GAL5, ST8SIA1, ST3GAL2*, and *NEU3* in adherent- or 3D-cultured cells with or without 1 μM PD0325901 treatment. Results shown are normalized to values obtained for adherent-cultured cells (value = 1). **p* < 0.05, ***p* < 0.01. n.s.: not significant.

**3. Supplementary western blot dataset**
